# Evidence for the Evolution of Resistance to Non‐Chemical Parasite Controls: Salmon Lice From Submerged Cages Produce Larvae That Swim Deeper

**DOI:** 10.1111/eva.70167

**Published:** 2025-10-07

**Authors:** Lowri Angharad O'Neill, Andrew Coates, Frode Oppedal, Tim Dempster

**Affiliations:** ^1^ Sustainable Aquaculture Laboratory, Temperate and Tropical (SALTT), School of Life and Environmental Sciences Deakin University Geelong Victoria Australia; ^2^ Population Biology and Genomics, School of Molecular and Life Sciences Curtin University Perth Western Australia Australia; ^3^ Institute of Marine Research, Matre Aquaculture Research Station Matredal Norway

**Keywords:** aquaculture, *Lepeophtheirus salmonis*, resistance, *Salmo salar*, sea lice, selection pressure

## Abstract

Salmon lice ( *Lepeophtheirus salmonis* ) pose a major challenge to the sustainability of salmon aquaculture due to their capacity to rapidly evolve resistance to parasite control methods. As the effectiveness of chemical treatments has declined, the industry has increasingly relied on preventive strategies to limit initial infections. One such approach is depth‐based farming, where fish are held deeper in the water column using submerged cages. These systems reduce exposure to lice, which typically concentrate near the surface. However, there is growing concern that such practices may inadvertently select for lice that are better adapted to deeper swimming, potentially enabling resistance to depth‐based interventions. In this study, we investigated whether vertical swimming behaviour in salmon lice larvae is influenced by the depth at which their parents were collected. We sampled 122 adult female lice carrying egg strings from commercial salmon farms using either standard cages (0–20 m) or submerged cages (20–40 m). The first‐generation larvae were reared under controlled conditions, and the vertical positioning of 11,291 copepodid larvae was tested in pressure columns simulating a depth of 10 m. Our results revealed a significant interaction between larval depth distribution and the cage type from which the parental lice were sourced (*χ* ^2^ = 278.85, df = 1, *p* < 0.001). Larvae from standard cages showed a greater tendency to ascend (35% vs. 23%) and were less likely to sink (19% vs. 27%) compared to larvae from submerged cages. These findings suggest that vertical swimming behaviour may be heritable, with submerged cages potentially selecting for deeper‐dwelling lice over time. This study provides the first evidence that the depth preference of salmon lice larvae may be influenced by their parents' environment. Understanding this behavioural inheritance is crucial for evaluating the long‐term sustainability of submerged cage systems and for developing lice management strategies that anticipate evolutionary responses.

## 1 Introduction

Aquaculture is experiencing rapid global expansion, often referred to as the ‘blue revolution’, with significant potential for further growth (Subasinghe et al. 1998). Among the key species driving expansion is the Atlantic salmon ( *Salmo salar* ), which is the most economically significant aquaculture species worldwide (Garlock et al. 2020; FAO 2024). Effective and sustainable strategies for parasite prevention and treatment are crucial to maintain current production levels. Since its inception in Norway in the late 1960s, salmon aquaculture has faced persistent challenges from infestations of the salmon louse ( *Lepeophtheirus salmonis* ; Torrissen et al. 2013). These infestations, along with associated diseases, represent major bottlenecks in advancing the blue revolution of aquaculture, posing long‐term problems for production and sustainability (Taranger et al. 2015).

Infestations can reduce the health and welfare of farmed salmon, leading to physical damage, skin erosion, reduced swimming speed, osmoregulation failure, secondary infections, immunosuppression, chronic stress and growth‐related mortality in salmonids (Bowers et al. 2000; Wagner et al. 2003; Grave et al. 2004; Hamre et al. 2009; Skilbrei et al. 2013; Bui et al. 2020). Furthermore, these infestations have spillback effects that negatively impact wild fish stocks (Bjørn et al. 2001; Torrissen et al. 2013; Kristoffersen et al. 2018). Consequently, regulations in several countries mandate anti‐lice treatments when infestations exceed specific thresholds. Farmed salmon represent > 99% of available hosts in Norway (Dempster et al. 2021) and are significant drivers of the ecology and evolution of salmon lice. This dominance has disrupted the balance of host–parasite seasonality, making farms hotspots for high lice density throughout the year. The dense concentration of hosts in aquaculture environments exerts evolutionary pressures on the parasite, leading to rapid changes in its biology and behaviour to adapt to these artificial conditions (Coates et al. 2023).

For decades, chemotherapeutic compounds dominated sea lice management and helped limit production losses in aquaculture, but the emergence of widespread resistance greatly reduced their effectiveness (Aaen et al. 2015). This prompted a shift toward non‐chemical approaches such as mechanical, thermal and biological delousing methods (Abolofia et al. 2017). These alternatives are often costly and have been linked to reduced growth and high mortality rates among salmonids, raising concerns over animal welfare (Overton et al. 2019). Moreover, laboratory studies have revealed variation in thermal and freshwater tolerance among lice populations, creating concerns about the long‐term effectiveness of these non‐chemical strategies (Ljungfeldt et al. 2017; Groner et al. 2019; Andrews and Horsberg 2020). Unlike chemical resistance, which can arise through relatively simple genetic mutations, resistance to non‐chemical controls may require more complex physiological, anatomical, or behavioural adaptations. These shifts could have broader ecological implications, potentially altering the ecological niche of salmon louse compared to what they encounter in nature through changes in salinity, temperature, or pressure preferences (Coates et al. 2021). Despite evidence of these challenges, the industry continues to rely heavily on short‐term control measures, which often lose effectiveness shortly after implementation (Brakstad et al. 2019). Growing concerns about resistance and sustainability in control methods are increasingly prompting a transition toward more preventative strategies.

Prevention‐based methods are the most effective and crucial first line of defence against sea lice infestations and prevent infestation from happening in the first place (Barrett et al. 2020; Jeong et al. 2021). Salmon lice have a complex life cycle, beginning with two nauplius stages before developing into the infective copepodid stage. Copepodid is the only stage responsible for infecting fish in cages (Pike and Wadsworth 1999). In the planktonic stages, larvae are highly motile, using environmental cues to position themselves in the water column and locate hosts (Johnson et al. 1996; Hamre et al. 2013). Innovative ‘depth‐based’ technologies, such as lice skirts, snorkel cages and deep‐sea feeding and lighting, are increasingly being adopted in aquaculture systems. These approaches aim to reduce lice infestations while avoiding costly delousing treatments and maintaining good fish welfare (Overton et al. 2019; Warren‐Myers et al. 2024). Lice skirts are physical mesh barriers around fish cages, preventing lice from entering (Stien et al. 2016; Jónsdóttir et al. 2023). Submerged cages keep fish deeper in the water column, where lice are less prevalent (Dempster et al. 2008, 2009; Korsøen et al. 2009; Oppedal et al. 2020; Warren‐Myers et al. 2022, 2024) and are now successfully used on a commercial scale (Lysø 2023; Thun and Guddingsmo 2024) at > 20 sites using > 160 cages (pers. Comm Frode Oppedal). Deep‐sea feeding and lighting methods provide food and light at lower depths, drawing fish away from the surface and encouraging them to stay deeper (Barrett et al. 2020; Bui et al. 2020). These technologies leverage the fact that planktonic copepodids primarily occur at the water surface (Crosbie et al. 2019), where lice are more likely to encounter wild salmonids, especially during migration or when filling their swim bladders, and are known to reduce infesations (Oppedal et al. 2017; Wright et al. 2017) without impacting fish welfare (Stien et al. 2018). By keeping fish deeper, the likelihood of parasite encounters is reduced (Geitung et al. 2019). While snorkel cages are limited in commercial use (Geitung et al. 2019), shielding skirts are among the most established barrier technologies (Barrett et al. 2020; Jónsdóttir et al. 2023). Submerged cages have entered production and reduce infestations by 72%–96% compared to surface cages (Sievers et al. 2019; Warren‐Myers et al. 2022). Despite strong efficacy in preventing salmon lice, most depth‐based technologies are not fully enclosed, allowing water flow and thus lice, into the cages. Some lice inhabit deeper waters naturally and infect fish in deeper cages (Frank et al. 2015) as illustrated by recent commercial experience (Thun and Guddingsmo 2024). The potential for lice to adapt to depth‐based farming, and the selective pressures this may place on traits such as depth preference, remains largely unexplored, likely due to the technology's relative infancy (Groner et al. 2016; Brakstad et al. 2019). This represents a key knowledge gap in parasite control within aquaculture, particularly as investment in preventative methods increases and technological development accelerates in response to new regulations regarding lice‐related production limits. The limited evidence on how lice may adapt to these interventions underscores the need for further research to ensure the long‐term effectiveness of control strategies. If adaptation occurs, it is likely to be gradual and complex (Coates et al. 2020), with the potential to alter the species' evolutionary trajectory, making this an especially important area of inquiry.

Substantial phenotypic variation in the swimming behaviour of salmon lice under pressure has been observed, suggesting that depth‐based technologies could impose selection on phenotypes that position lice deeper in the water column (Coates et al. 2020). However, this study focused on within‐family variation and found no evidence that these behavioural traits are heritable or shaped by selection, leaving open the question of whether such traits could persist across generations. It remains unclear whether deeper‐swimming behaviours can be passed down to offspring, or if parental generations exert any influence on the hydrostatic response of larvae. Understanding this potential for heritable change is essential for predicting the long‐term effectiveness of depth‐based control strategies in aquaculture. Evolutionary adaptations in depth distribution have been documented in other planktonic crustaceans, where inherited traits influence vertical positioning. For example, Daphnia species show genotype‐specific depth distributions in response to chemical cues from predators (De Meester 1993, 1996), and Calanoid copepods exhibit depth‐related variation linked to lipid content, affecting buoyancy and vertical positioning (Zarubin et al. 2014). Although rare, pest adaptation to non‐chemical preventative controls has been observed in terrestrial systems. In the western corn rootworm ( *Diabrotica virgifera* ), crop rotation with soybeans initially disrupted its life cycle, but by 1995, damage reappeared in rotated fields. The rootworms had evolved the ability to digest soybean defences, effectively bypassing rotation and demonstrating that pests can adapt to overcome preventative strategies (Gray et al. 2009). These examples underscore how heritable traits can shape behavioural responses to preventative strategies over time and highlight the possibility that salmon lice could also adapt to deep farming methods, although this has yet to be demonstrated.

In this study, we examined differences in the vertical movements of copepodid larvae hatched from egg strings collected from adult lice sampled from standard and submerged salmon cages. We hypothesised that copepodids from standard cages would show a stronger response to hydrostatic pressure by swimming toward the surface, while those from submerged cages would have a weaker response and be more likely to sink. Hydrostatic pressure increases with depth and can influence the behaviour and vertical distribution of copepodid larvae (Coates et al. 2020), but it remains unclear whether this response is influenced by parental origin or passed down to offspring. To investigate this, we conducted laboratory experiments using pressurised water columns to simulate ambient pressure at 10 m. This setup allowed us to test if parental origin, whether from standard or submerged cages, affects the vertical distribution and swimming behaviour of larvae. The results could provide insight into whether submerged cages could select for genotypes that favour deeper‐swimming behaviour, potentially influencing the evolutionary niche of pressure preference in sea lice populations and the long‐term effectiveness of depth‐based preventative technologies. This question is crucial for the future of sustainable aquaculture.

## 2 Methods

### 2.1 Louse Collection and Larval Production

**FIGURE 1:**
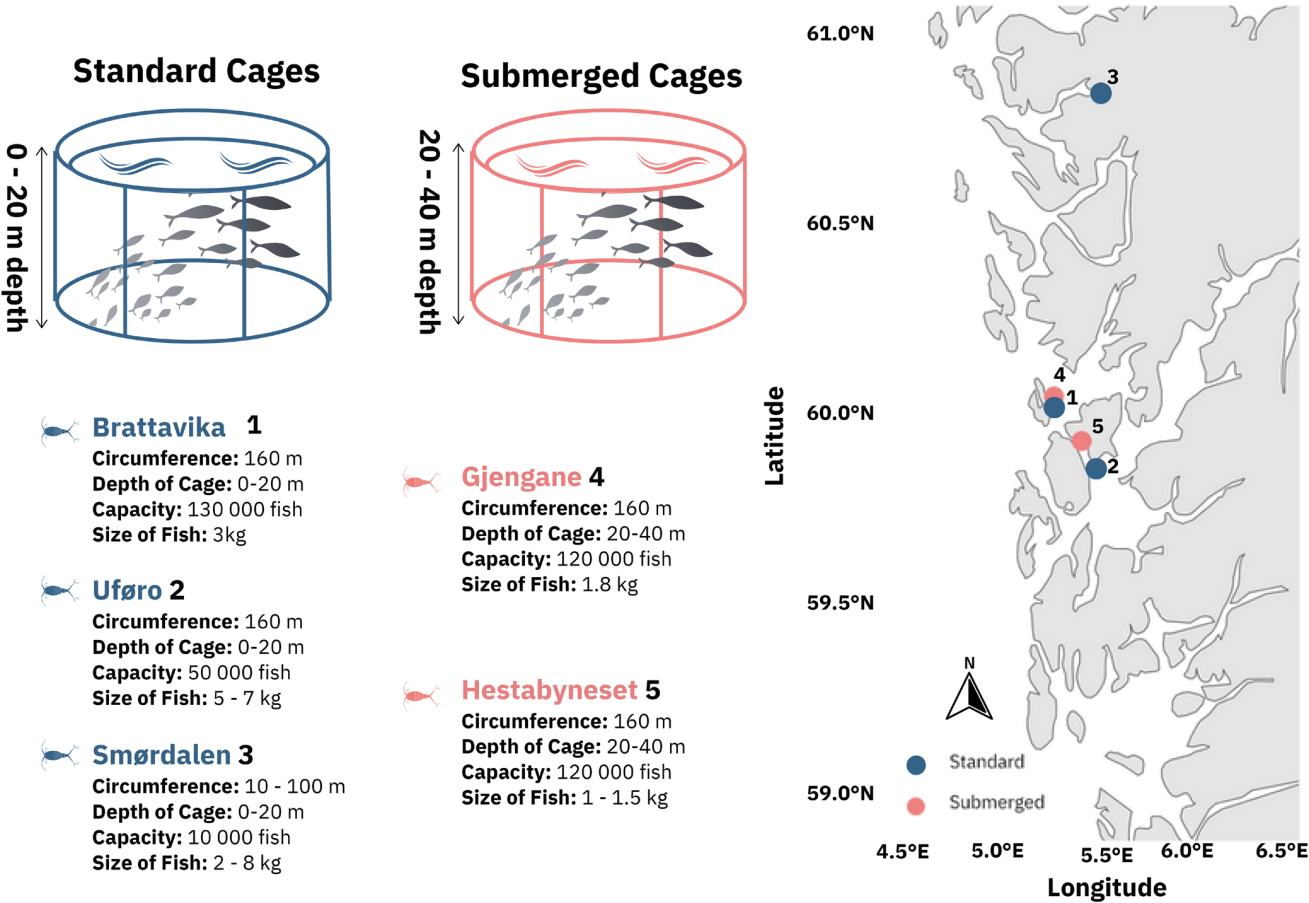
Collection locations and their characteristics. Blue points represent standard cages, with main water depth available to the fish at 0–20 m deep, while red points indicate submerged cages with an airdome positioned in the centre, with main depth at 20–40 m deep. All cages are located in the Karmøy to Sotra major production zone, except for Smørdalen, which is situated furthest north in the Nordhordaland to Stadt region.

From early April to late May 2024, we collected female *L. salmonis* from five salmon farms during routine lice counts within all cages at the sites (Figure 1). We gathered 25 pairs of egg strings from adult females at two standard Atlantic salmon cages in Smørdalen and Brattavika, and 22 pairs from a standard cage in Uforø. For the submerged farms at Gjengane and Hestabyneset, we collected lice from recently deceased fish, as live fish sampling before harvest is difficult in submerged technology. The dead fish sank to the bottom of the submerged cages, where a lift‐up suction pump brought them to the surface. The fish were then placed into a bin with a grid to allow water drainage, making it easy to collect still‐attached lice. We obtained 25 pairs of egg strings from Gjengane and 11 pairs of egg strings from Hestabyneset, as well as 14 females and 4 males for laboratory reproduction.

After collection, lice were transported in chilled seawater to the Institute of Marine Research's station in Matre, Masfjorden. Upon arrival, egg strings were carefully removed from the lice using tweezers. Each pair of egg strings from a female adult was incubated separately under controlled conditions (13°C, 34 PSU), with daily monitoring of development.

Twelve salmon of about 400 g, approximately 2 years old and from the Aquagen strain, were sourced from holding tanks at the station. These salmon were placed in 400 L tanks at the research station and maintained at 12°C and 34 PSU for 2 months. Adult lice (both male and female) from submerged cages at Hestabyneset were transferred onto these salmon under controlled conditions. The fish were sedated with Metomidate Hydrochloride (0.01 g/L, C_13_H_15_ClN_2_O), and the lice were applied to the fish's skin using tweezers and waterproof paper. The lice were monitored until new egg strings were produced. Once this occurred, the salmon were sedated again, and the egg strings were carefully removed and incubated. All egg strings, whether collected directly from farms or produced in the lab, were incubated under the same controlled conditions (13°C, 34 PSU) until they developed into copepodids. In total, egg strings from 122 individual females were used in the pressurised column experiments: 108 collected directly from standard and submerged farms, and 14 produced in the lab from adult lice collected at Hestabyneset (submerged cage). These additional egg strings were produced to supplement the larvae from Hestabyneset, where egg string availability was insufficient.

For the experiments, we used 3‐day‐old copepodids, the stage at which they are most infectious (Tucker et al. 2000; Skern‐Mauritzen et al. 2020). Due to natural variation in the level of egg string maturity, the copepodids hatched in a staggered manner. In total, 11,291 copepodids were assayed to investigate their vertical distribution in response to hydrostatic pressure, with a focus on how their swimming behaviour might be influenced by the parental generation, either from standard or submerged cages.

### 2.2 Experimental Columns

**FIGURE 2:**
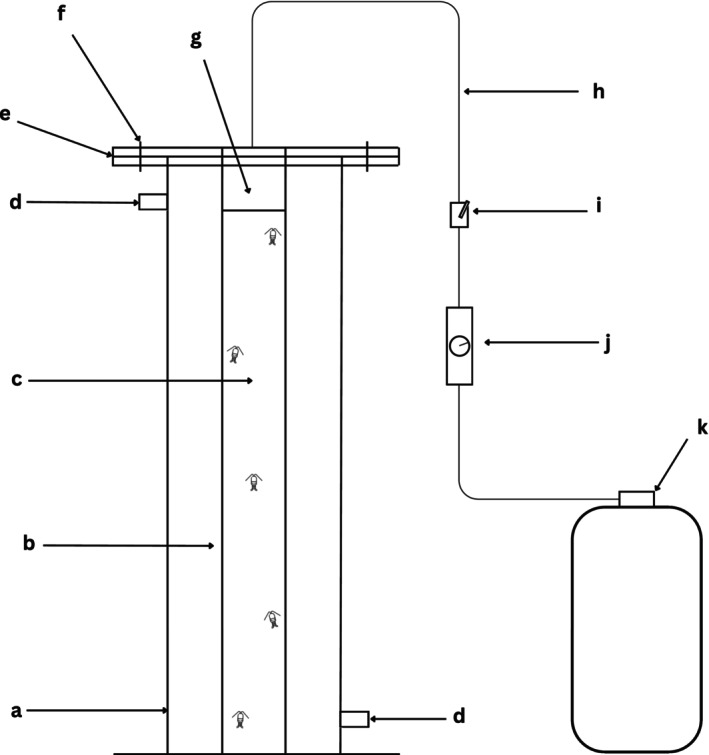
Experimental column used to test the vertical distribution of copepodid larvae from standard and submerged cages. Components include: (a) water tank acting as a water bath to maintain a constant temperature within the tube, (b) PVC tube, (c) testing tube for lice, (d) water inflow and outflow, (e) lid covering the apparatus, (f) holes for securing the lid with nuts and bolts, (g) lice entry point, (h) hose connecting the scuba tank to pressurise the column, (i) adjustable regulator, (j) pressure reducer and (k) scuba tank (following Coates et al. 2020).

Experiments were conducted using small‐scale water columns, as illustrated in the schematic diagram in Figure 2, following procedures established by Coates et al. (2020) and Crosbie et al. (2019, 2020). Each column consisted of a clear polyvinyl chloride (PVC) tube, 85 cm in height and 4.5 cm in internal diameter, positioned vertically and sealed at the bottom. Prior to each experiment, the tube was filled with seawater to a height of 80 cm, with a salinity of 34 PSU. The tube was then placed inside a transparent PVC box (85 cm × 20 cm × 20 cm) acting as a water bath maintained at a constant temperature of 13°C. A white backdrop behind the column was used in order for there to be enough contrast to be able to see the copepodids clearly with the naked eye. To minimise light exposure, the area was surrounded by black plastic panels, keeping the experimental space dark. A PVC lid (28 cm^2^) was securely fastened over the apparatus with bolts, creating a 5 cm air pocket above the water. Rubber O‐rings ensured an airtight seal. A 6 mm hose, inserted through a hole in the lid, connected the column to a scuba tank of compressed air via a pressure reducer and adjustable regulator (Festo LFR‐D‐MINI‐A, Festo, Australia). The scuba tank introduced air to pressurise the air pocket, increasing the hydrostatic pressure of the seawater (Stake and Sammarco 2003). Desired pressure levels were achieved in less than 4 s, after which no additional air was introduced or lost. To validate the apparatus, an inverted graduated test tube containing an air bubble was submerged in the column. When 1 bar of additional pressure was applied, the air bubble compressed to half its original volume, consistent with Boyle's law.

### 2.3 Experimental Assays

The swimming behaviour of 3‐day‐old infectious copepodids (all lice within a single replicate came from the same egg string) was tested under 2 atm of pressure (ambient pressure at 10 m depth) for a 5‐min interval. This duration was chosen based on research by Coates et al. (2020), as it captures the maximum behavioural response after which little to no further change was observed in the vertical distribution of copepodids.

Before each experiment, the tube was filled halfway with seawater at 34 PSU. Live lice were carefully transferred into the tube using a pipette to ensure deceased specimens or any viscous residues at the bottom of the incubator were not added. The remaining volume of the tube was then filled with seawater, allowing the lice to mix evenly throughout the height of the column. After being transferred, the lice were left to acclimatise in darkness for 15 min. All columns were run in darkness (underwater light levels = 0.006–0.03 μmol m^2^ s^1^, measured with a LI‐COR LI‐1500 light sensor, LI‐COR Biosciences, Germany). After 5 min of pressurisation, the lice distribution was recorded on a sheet of paper stuck to the front of the column with water. With the aid of a head torch, larvae were visible to the naked eye. The water level in the tube was marked, and the distribution of lice was recorded on the sheet at 10 cm intervals from 10 cm to 80 cm. The counting was done within 45 s to minimise the effect of the head torch light on the lice distribution (Szetey et al. 2021). The same scientist recorded the vertical distribution each time to avoid sampling bias. In total, 122 columns were run (25 columns each for 5 locations, excluding 3 from one location) over a period of 2 months.

### 2.4 Statistical Analyses

First, we assessed whether the source of the eggs from lice sampled from Hestabyneset (whether collected directly from the submerged cage or produced after lice were transferred to the laboratory) influenced the results. We tested this using a Mann–Whitney *U*‐Test. The interaction between depth and egg string source (submerged farm vs. laboratory‐reared) was not significant (*W* = 11,808, *p* = 1), and patterns of distribution with depth were similar (see Figure S1). Larvae from both sources for Hestabyneset were included in subsequent analyses.

To analyse the vertical distribution of copepodid‐stage salmon lice larvae, we calculated the proportion of larvae observed at each 10 cm depth interval within the experimental columns. The distribution was bimodal, with higher proportions at the surface (0–10 cm; hereafter 10 cm) and near the bottom (70–80 cm; hereafter 80 cm), which accounted for 53% of the observed lice across trials. Intermediate depths (10–70 cm) showed minimal variation and were excluded from further analysis (as per Coates et al. 2020).

A generalised linear model (GLM) with a binomial distribution was initially used to test the effect of cage type (submerged vs. standard) on vertical swimming behaviour at 10 cm and 80 cm. However, model diagnostics revealed significant overdispersion (dispersion ratio = 8.17), indicating that the variance exceeded what a standard binomial model could account for. To correct for this, we refitted the model using a quasibinomial distribution, which accounts for overdispersion and provides more reliable standard errors and significance testing. All analyses were performed in R version 4.3.1 (2023‐06‐16).

## 3 Results

**FIGURE 3:**
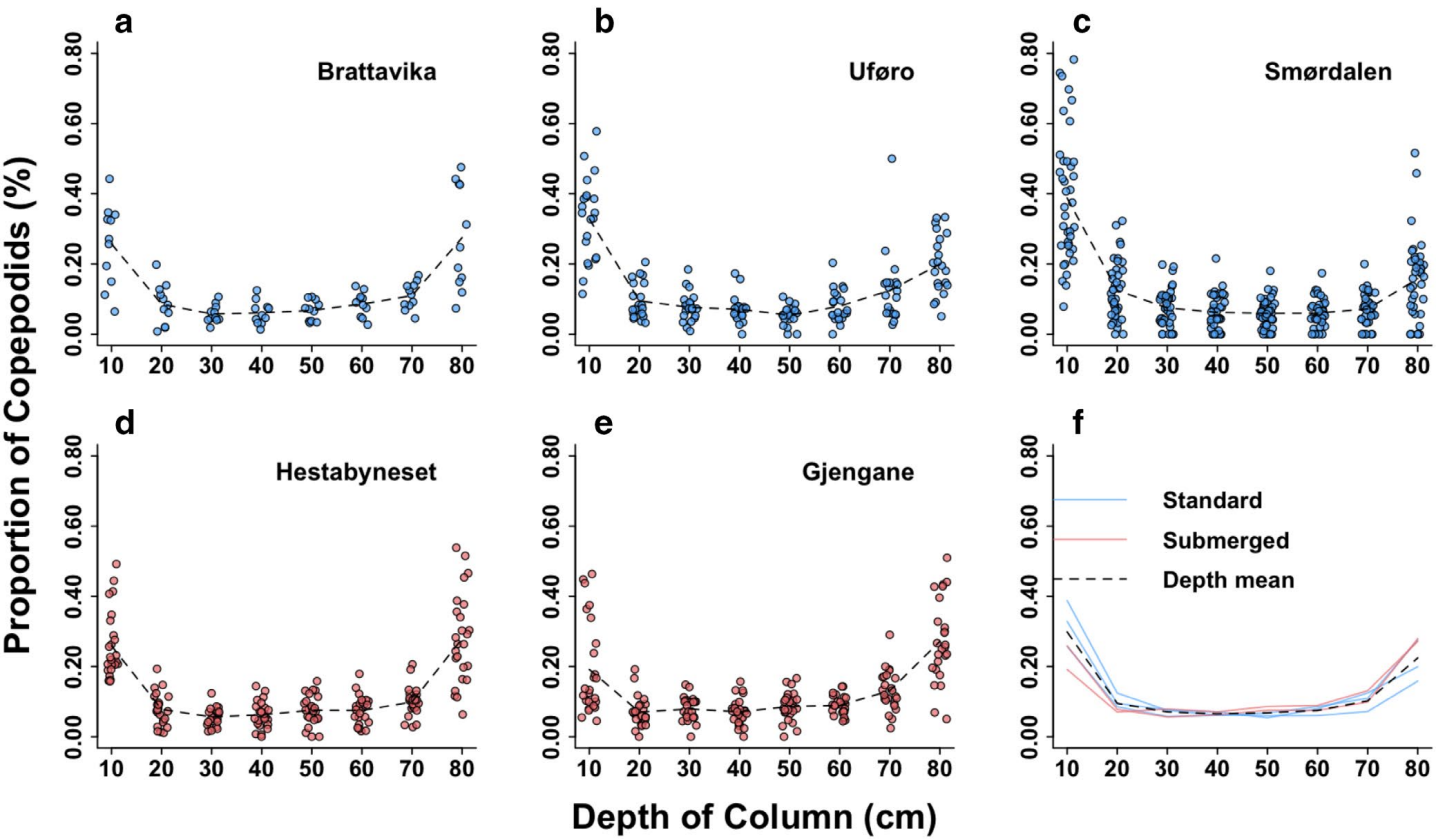
Proportion of copepodids across 10 cm to 80 cm depths, measured in 10 cm intervals, after 5 min of exposure to 2 atm pressure (simulated 10–10.8 m depth), categorised by parental origin (standard cages in blue, submerged cages in red). Panels a–c show copepodid proportions from standard cages: (a) Brattavika, (b) Uføro, (c) Smørdalen. Panels (d, e) show proportions from submerged cages: (d) Hestabyneset, (e) Gjengane. Panel (f) summarises the mean proportion per depth for each cage. Note Brattavika (blue) and Hestabyneset (red) both start at the same proportion (0.25) on the y‐axis and overlap at that point. The black dotted lines in panels (a–e) represent cage‐wise mean proportions at each depth, and the black dotted line in panel F represents the mean proportion of copepods across both cages at each depth.

**FIGURE 4:**
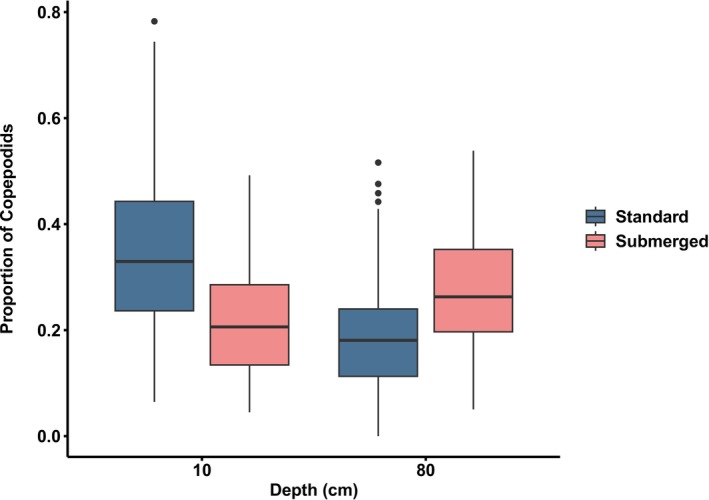
Mean proportion of copepodids at 10 cm (top) and 80 cm (bottom) depths after 5 min under 2 atm pressure. Blue boxes represent proportions of larvae at 10 cm and 80 cm sourced from parents collected from standard cages, while red boxes represent proportions of larvae sourced from parents collected from submerged cages at these depths. The boxplot illustrates the data distribution, showing the median (line inside the box), the interquartile range (IQR, represented by the box) and potential outliers (points outside 1.5 times the IQR, plotted as individual dots).

After being exposed to 2 atm of pressure for 5 min (simulating a depth of 10–10.8 m), copepodids exhibited a bimodal depth distribution. Less than 10% were observed at depths of 20–70 cm (Figure 3), while 5%–50% were found at 10 and 80 cm. A GLM with a quasibinomial error distribution revealed a highly significant interaction between cage type and depth (*χ* ^2^ = 278.85, df = 1, *p* < 0.001). Standard cages had a higher proportion of larvae at 10 cm (*F*(1, 119) = 21.2, *p* < 0.001), while submerged cages had a higher proportion at 80 cm (*F*(1, 120) = 15.6, *p* < 0.001; Figure 4). For larvae from standard cages, the median proportion at 10 cm was 0.35 ± 0.16 SD, while for submerged cages this was only 0.23 ± 0.12. For the 80 cm depth, the median proportion of larvae from standard cages was 0.19 ± 0.12, while it was 0.27 ± 0.12 for submerged cages (Figure 4).

## 4 Discussion

Our study demonstrates that the vertical distribution of salmon lice larvae differs significantly based on the cage type occupied by their parental generation, with lice in submerged cages producing offspring with a stronger tendency to sink compared to those from standard cages. The consistent behavioural patterns of less upward movement at the 10 m depth pressure level suggest that parental origin influences the response of offspring to hydrostatic pressure in natural environments. This raises the possibility that depth‐based prevention methods favour the selection of deeper‐swimming louse genotypes, which may have an improved capacity to infect salmon in submerged cages. However, further research is needed to determine whether these traits are heritable, how prevalent they may become, and their potential impact on the long‐term effectiveness of submerged cages.

The observed bimodal swimming behaviour, characterised by intermittent upward swimming followed by passive sinking, aligns with patterns seen in previous studies (Crosbie et al. 2019, 2020; Szetey et al. 2021; Coates et al. 2020) and in other planktonic crustaceans (Heuch 1995; Flamarique et al. 2000). The vertical distributions of plankton are driven by environmental pressures such as food availability and predator evasion (Gaines and Bertness 1992), while in the case of lice, host availability plays a key role. It is possible that swimming behaviour in salmon lice is also influenced by chemical cues from salmon (Devine et al. 2000) or conspecifics (Morefield and Hamlin 2022). This may be particularly relevant in deeper environments, where host interactions are less common for the lice and may present challenges, as they are not typically exposed to these conditions in surface waters.

Our findings build on those of Coates et al. (2020), who observed within‐family variation in copepodid vertical distribution in standard cages, suggesting strong selection potential in swimming behaviour. However, Coates did not find evidence of this trait being passed down to offspring, nor did offspring exhibit the same behaviours as their parents. In contrast, our study differs by examining lice from different environmental conditions: those exposed to standard cages (0–20 m) and those exposed to submerged cages (20–40 m). We observed similar deep‐swimming behaviours in the larvae of the lice collected from submerged cages, strongly suggesting that these behaviours were inherited across generations. While our study did not explicitly investigate genetic inheritance, the consistency of the behavioural patterns, where larvae from standard cages consistently ascended under pressure and larvae from submerged cages consistently sank, indicates that this is more than just within‐family variation. The large sample size and repeated behaviour patterns strongly imply that these traits are likely being passed down to offspring, highlighting the need for further investigation into the heritability of these behaviours. Our findings thus expand on Coates' work by suggesting that this vertical swimming behaviour may be inherited, offering a deeper understanding of how these adaptations could shape sea lice behaviour over time. Depth‐based adaptations are a common evolutionary response among copepods. For example, depth preference among *Daphnia* is driven by predatory pressures and chemical cues (Dumont et al. 1985; De Meester 1993; King and Miracle 1995), and *Cyclops* species are thought to have developed migration strategies to evade predators (Gliwicz 1986). This suggests that, similarly, salmon lice may evolve depth‐related behaviours to better infest salmon at deeper depths, where they could avoid surface predators or find more suitable hosts.

No significant differences were found between egg strings collected directly from gravid females on submerged farms and those from females transferred to laboratory conditions (Figure S1). This indicates that the observed behavioural differences were not due to eggs experiencing different environmental conditions in submerged cages, and that host condition (being dead or alive when egg strings were removed) did not affect larvae in this context. Additionally, lice can survive off hosts for extended periods (Dalvin et al. 2025), so energy reserves are unlikely to be affected within 24 h; the daily collection of dead fish from submerged cages minimises potential impacts on larvae condition. While both sets of submerged egg strings and egg strings from standard cages were incubated under consistent laboratory conditions until they hatched to reduce environmental variation, maternal factors such as yolk reserves may still influence phenotypic traits, including buoyancy and depth preferences (Campbell and Dower 2003; Zarubin et al. 2014; Mennerat et al. 2017). Multi‐generational experiments under standardised rearing conditions would be necessary to fully disentangle maternal, environmental, and genetic effects, though such studies remain rare for salmon lice (Bakke et al. 2018).

The dispersal and vertical distribution of salmon louse larvae are influenced by environmental conditions, with hydrodynamic effects playing a crucial role. Larvae tend to sink in response to increased freshwater inflow in fjords to avoid brackish layers or excessive turbulence (Heuch 1995; Crosbie et al. 2019, Heuch et al. 2009), resulting in natural variation in their vertical distribution within the water column. Our observations show that a portion of lice from both standard and submerged cages consistently sank to the bottom of the column under pressure conditions simulating a depth of 10 m. This suggests that, in natural settings, these lice may remain at or sink beyond this depth before responding to pressure cues. As lice found at greater depths are more likely to infect fish in submerged cages, which are typically positioned below 20 m, this sinking behaviour likely has implications for infection risk (Frank et al. 2015; Samsing et al. 2016; Bui et al. 2020). Although our columns were limited to simulating 10 m depth, the consistent downward movement of some lice across all replicates suggests they may have continued sinking, potentially reaching depths sufficient to infect fish in submerged systems.

As a result, sea lice populations in submerged cages, as well as in cages that use depth‐based control strategies such as skirts or deep‐feeding, may become increasingly composed of individuals with a behavioural tendency to remain at greater depths. Over time, this could lead to a shift in the overall depth preference of the population. This process may be reinforced through spatial sorting, an evolutionary mechanism in which individuals with similar traits, such as a preference for deeper water, are more likely to encounter and mate with each other (Phillips and Perkins 2019). Repeated generations of such assortative mating could increase the prevalence of deeper‐dwelling phenotypes within the local sea lice population. The similarity in copepodid depth preferences across multiple submerged farms, even those over 27 km apart (Karmøy to Sotra), is consistent with the high connectivity and genetic mixing expected between farms within a major production zone. This consistency indicates that deeper‐swimming phenotypes may already be widespread. Given the high density of salmon in both standard and submerged cages, combined with their ability to move vertically within the water column, lice adapted to deeper environments could easily infest both cage types. If these traits are passed down across generations, they may reduce the effectiveness of depth‐based farming strategies over time.

Farmed salmon have a significant impact on the ecology of salmon lice (Dempster et al. 2021), influencing both their dispersal patterns and interactions with wild hosts (Coates et al. 2020). Currents that drive plankton transport vary in direction and strength with depth (Johnsen et al. 2016; Samsing et al. 2015; Samsing et al. 2016; Garnier et al. 2024), shaping the horizontal and vertical distribution of copepodids. Adaptations for deeper swimming could shift the spatial niche of lice, bringing them into closer alignment with hosts in deeper farming environments, while potentially reducing their contact with wild salmon at the surface (Coates et al. 2021). However, these adaptations may come with trade‐offs, such as reduced access to wild or farmed hosts that occupy shallower waters. It remains unclear whether such shifts in depth preference would significantly reduce lice transmission to wild populations over the long term. Nonetheless, if lice increasingly occupy deeper parts of the water column, this behavioural change could offer some benefit to wild salmon by reducing their exposure to infective stages. Deeper farming systems that modify the behavioural phenotype of salmon lice are likely to influence the ecological and evolutionary trajectories of sea lice populations. Similar adaptive responses to environmental pressures have been documented in other pests, such as the corn rootworm beetle, which evolved resistance to crop rotation (Levine et al. 2002). In this case, rootworms developed the ability to digest soybean defences, effectively circumventing crop rotation and demonstrating that pests can adapt to overcome preventative measures (Gray et al. 2009). These examples highlight how heritable traits can drive behavioural changes in response to management strategies over time and emphasise the potential for marine pests to follow similar adaptive trends observed in terrestrial systems, often emerging years later in aquaculture. This includes resistance to chemotherapeutants, non‐chemical treatments and other preventative methods.

The Atlantic population of salmon lice is panmictic (Glover et al. 2011), meaning beneficial mutations can spread rapidly across regions. If sufficient genetic variation and heritability exist, traits that enhance survival, such as deeper swimming, could become more widespread over time (Glover et al. 2011; Besnier et al. 2015; Coates et al. 2023). This study provides evidence that depth‐based resistance is possible. A clear example of rapid adaptation is the evolution of chemotherapeutant resistance, which is driven by single‐point mutations that can quickly spread across the Atlantic population (Coates et al. 2023).

However, resistance to depth‐based strategies is unlikely to evolve as quickly. Submerged cages are not used exclusively and are typically combined with other treatments, reducing consistent selection pressure. Unlike chemotherapeutant resistance, which can arise from a single genetic mutation, resistance to submerged cages would require a change in behavioural phenotype, which is more complex and slower to develop (Coates et al. 2021). Despite the potential for adaptation, depth‐based technologies such as submerged cages offer significant benefits for salmon welfare, productivity and environmental impact (Barrett et al. 2020; Warren‐Myers et al. 2024). Preserving the long‐term effectiveness of these strategies is important. Future research should focus on understanding the genetic basis of behavioural adaptations through multi‐generational studies, including seasonal replication and using in situ column trials that better reflect natural conditions (Tang et al. 2011).

Synergistic and antagonistic management strategies can improve sea lice control by slowing or halting the evolution of resistance (McEwan et al. 2016). Synergistic methods apply complementary selection pressures that target lice more effectively, while antagonistic strategies impose opposing pressures that limit resistance, like a surface barrier to restrict lice from sinking and infecting fish in submerged cages. Combining these approaches may reduce the likelihood of lice developing adaptations that reduce the efficacy of treatments (Coates et al. 2021). Further research is needed to understand how different management strategies influence lice evolution. While current evidence is insufficient to identify counterstrategies, balancing selection pressures should be a priority to maintain the longevity of preventative measures. Investigating how these pressures interact and affect lice phenotypes can help design approaches that minimise resistance. A comprehensive understanding of these evolutionary dynamics is essential for sustainable sea lice management.

## 5 Conclusion

Our findings demonstrate that salmon lice larvae exhibit vertical movement patterns that reflect the depth environment of their parental origin. Specifically, larvae from lice collected in submerged cages showed a weaker hydrostatic response, tending to sink more under pressure, while those from standard cages displayed a stronger response, actively swimming toward the surface. These consistent behavioural differences suggest that vertical swimming behaviour and depth preference are traits that can be passed from parent to offspring. Although the precise genetic basis for this behaviour is not yet understood, our results provide compelling evidence for its heritability. This raises important questions about the potential for sea lice populations to gradually adapt to depth‐based control strategies such as submerged cages, skirts and deep‐feeding systems. Understanding both the genetic and environmental factors that shape vertical distribution is therefore essential for anticipating the long‐term effectiveness of these technologies. Continued research is needed to explore the mechanisms underlying these behaviours, including multi‐generational studies and field trials that reflect natural conditions. A better grasp of how lice respond to vertical selection pressures will help inform the development of more resilient and sustainable sea lice management approaches.

## Ethics Statement

Ethics for holding lice on fish in the lab and fish sedation were approved by the Norwegian Animal Ethics Committee (FOTS no. 29848).

## Conflicts of Interest

The authors declare no conflicts of interest.

## Supporting information


**Figure S1:** Proportion of copepodids across 10–80 cm depths, measured in 10 cm intervals, after 5 min of exposure to an increase in hydrostatic pressure (from 1 to 2 atm). The data compares copepodids hatched from egg strings collected from submerged farms (blue) with those produced in laboratory conditions using females and males from submerged farms (red).

## Data Availability

Data on the vertical distribution of copepodids from lice in both standard and submerged cages has been uploaded and is openly available in the Dryad repository (https://doi.org/10.5061/dryad.b5mkkwhs6). Benefits from this research derive from the open sharing of data through a public repository (see above). Results of this study are accessible to the broader scientific community and stakeholders concerned with salmon aquaculture and parasite management.
