# Supplementary material for: Evidence for the Evolution of Resistance to Non‐Chemical Parasite Controls: Salmon Lice From Submerged Cages Produce Larvae That Swim Deeper
Source: Evol Appl. 2025 Oct 7;18(10):e70167. doi: 10.1111/eva.70167 (PMC12504137; doi:10.1111/eva.70167)
Supplement: Supplementary file 1 — Figure S1: Proportion of copepodids across 10–80 cm depths, measured in 10 cm intervals, after 5 min of exposure to an increase in hydrostatic pressure (from 1 to 2 atm). The data compares copepodids hatched from egg strings collected from submerged farms (blue) with those produced in laboratory conditions using females and males from submerged farms (red). [file EVA-18-e70167-s001.docx]

**Supplementary Materials**

***Supplementary Fig 1.***Proportion of copepodids across 10 cm to 80 cm depths, measured in 10 cm intervals, after 5 minutes of exposure to an increase in hydrostatic pressure (from 1 to 2 atm). The data compares copepodids hatched from egg strings collected from submerged farms (blue) with those produced in laboratory conditions using females and males from submerged farms (red).
